# Targeted deletion of the C-terminus of the mouse adenomatous polyposis coli tumor suppressor results in neurologic phenotypes related to schizophrenia

**DOI:** 10.1186/1756-6606-7-21

**Published:** 2014-03-29

**Authors:** Takanori Onouchi, Katsunori Kobayashi, Kazuyoshi Sakai, Atsushi Shimomura, Ron Smits, Chiho Sumi-Ichinose, Masafumi Kurosumi, Keizo Takao, Ryuji Nomura, Akiko Iizuka-Kogo, Hidenori Suzuki, Kazunao Kondo, Tetsu Akiyama, Tsuyoshi Miyakawa, Riccardo Fodde, Takao Senda

**Affiliations:** 1 Department of Anatomy I, Fujita Health University School of Medicine, Toyoake, Aichi 470-1192, Japan; 2 Department of Pharmacology, Graduate School of Medicine, Nippon Medical School, 1-1-5 Sendagi, Bunkyo-ku, Tokyo 113-8602, Japan; 3 Japan Science and Technology Agency, Core Research for Evolutional Science and Technology, Saitama 332-0012, Japan; 4 Department of Anatomy, Faculty of Clinical Engineering, Fujita Health University School of Health Sciences, Toyoake, Aichi 470-1192, Japan; 5 Department of Gastroenterology and Hepatology, Erasmus Medical Center, Rotterdam, The Netherlands; 6 Department of Pharmacology, Fujita Health University School of Medicine, Toyoake, Aichi 470-1192, Japan; 7 Department of Pathology, Saitama Cancer Center, Ina, Saitama 362-0806, Japan; 8 Center for Genetic Analysis of Behavior, National Institute for Physiological Sciences, Myodaiji, Okazaki, Aichi 444-8585, Japan; 9 Division of Systems Medical Science, Institute for Comprehensive Medical Science, Fujita Health University, Toyoake, Aichi 470-1192, Japan; 10 Genetic Engineering and Functional Genomics Group, Frontier Technology Center, Graduate School of Medicine, Kyoto University, Kyoto 606-8501, Japan; 11 Laboratory of Molecular and Genetic Information, Institute for Molecular and Cellular Biosciences, University of Tokyo, Bunkyo-ku, Tokyo 113-0032, Japan; 12 Department of Pathology, Josephine Nefkens Institute, Erasmus Medical Center, Rotterdam, The Netherlands; 13 Present address: Department of Pathology I, Fujita Health University School of Medicine, Toyoake, Aichi 470-1192, Japan; 14 Present address: Department of Anatomy and Cell Biology, Gunma University Graduate School of Medicine, Maebashi 371-8511, Japan; 15 Present address: Department of Anatomy, Gifu University Graduate School of Medicine, Gifu 501-1194, Japan

**Keywords:** *Adenomatous polyposis coli* (*Apc*), *Apc*^1638T/1638T^ mice, Hippocampus, Working memory, Locomotor activity, Schizophrenia

## Abstract

**Background:**

Loss of *adenomatous polyposis coli* (*APC*) gene function results in constitutive activation of the canonical Wnt pathway and represents the main initiating and rate-limiting event in colorectal tumorigenesis. APC is likely to participate in a wide spectrum of biological functions via its different functional domains and is abundantly expressed in the brain as well as in peripheral tissues. However, the neuronal function of APC is poorly understood. To investigate the functional role of Apc in the central nervous system, we analyzed the neurological phenotypes of *Apc*^1638T/1638T^ mice, which carry a targeted deletion of the 3′ terminal third of *Apc* that does not affect Wnt signaling.

**Results:**

A series of behavioral tests revealed a working memory deficit, increased locomotor activity, reduced anxiety-related behavior, and mildly decreased social interaction in *Apc*^1638T/1638T^ mice. *Apc*^1638T/1638T^ mice showed abnormal morphology of the dendritic spines and impaired long-term potentiation of synaptic transmission in the hippocampal CA1 region. Moreover, *Apc*^*1638T/1638T*^ mice showed abnormal dopamine and serotonin distribution in the brain. Some of these behavioral and neuronal phenotypes are related to symptoms and endophenotypes of schizophrenia.

**Conclusions:**

Our results demonstrate that the C-terminus of the Apc tumor suppressor plays a critical role in cognitive and neuropsychiatric functioning. This finding suggests a potential functional link between the C-terminus of APC and pathologies of the central nervous system.

## Background

Mutations of the tumor suppressor gene *adenomatous polyposis coli* (*APC*) are responsible for familial adenomatous polyposis (FAP) and sporadic colorectal cancers [1]. APC functions as an intracellular regulator of Wnt/β-catenin signal transduction, which is thought to represent its main tumor suppressing activity [1]. In addition to peripheral tissues, APC is broadly expressed in the central nervous system including the hippocampus and cerebral cortex in adult mammals [2]. However, except for the observed low penetrance of medulloblastoma among patients with FAP [3], *APC* mutations do not seem to be associated with brain tumorigenesis. APC is a large multifunctional protein encompassing several different functional domains, and its carboxy (C)-terminal domains are known to bind postsynaptic density-95 (PSD-95), end-binding protein 1, and microtubules [4]. APC is likely to participate in a wide spectrum of biological functions in addition to its known role in binding and regulating β-catenin [5]. It has been reported that *APC* is associated with the susceptibility for psychiatric disorders such as depression [6] and schizophrenia [7]. These lines of evidence suggest a potential role for APC in regulating neuronal functions in the brain. However, neurological or neuropsychological functions of APC have not been well investigated.

Several *Apc*-mutant mouse models have been generated that mimic the germline and sporadic mutations found in FAP and colon cancers [8]. Most of these mutant *Apc* genes encode truncated Apc proteins lacking the β-catenin-binding motifs and the C-terminal domains [1]. Because of the central role of Wnt signaling during development, most *Apc*–mutant mice are embryonic lethal when bred to homozygosity. Although heterozygous mice are viable, they develop multiple intestinal tumors [8], which may complicate the interpretation of the behavioral phenotype of the mutant mice [9]. One notable exception is the *Apc*^1638T^ mutation that encodes a truncated Apc protein lacking the C-terminal domains [10]. Since this mutation does not affect the β-catenin-regulating functional motifs, the β-catenin signaling assessed in mouse embryonic fibroblasts and embryonic stem cells remains intact [10]. Accordingly, *Apc*^1638T/1638T^ mice are viable and tumor-free [10]. In the present study, we examined the behavioral and neuronal phenotypes of *Apc*^1638T/1638T^ mice to elucidate the functional role of the C-terminus of Apc tumor suppressor in the central nervous system.

## Results

### Impaired learning and memory, and increased locomotor activity in *Apc*^*1638T/1638T*^ mice

**Figure 1:**
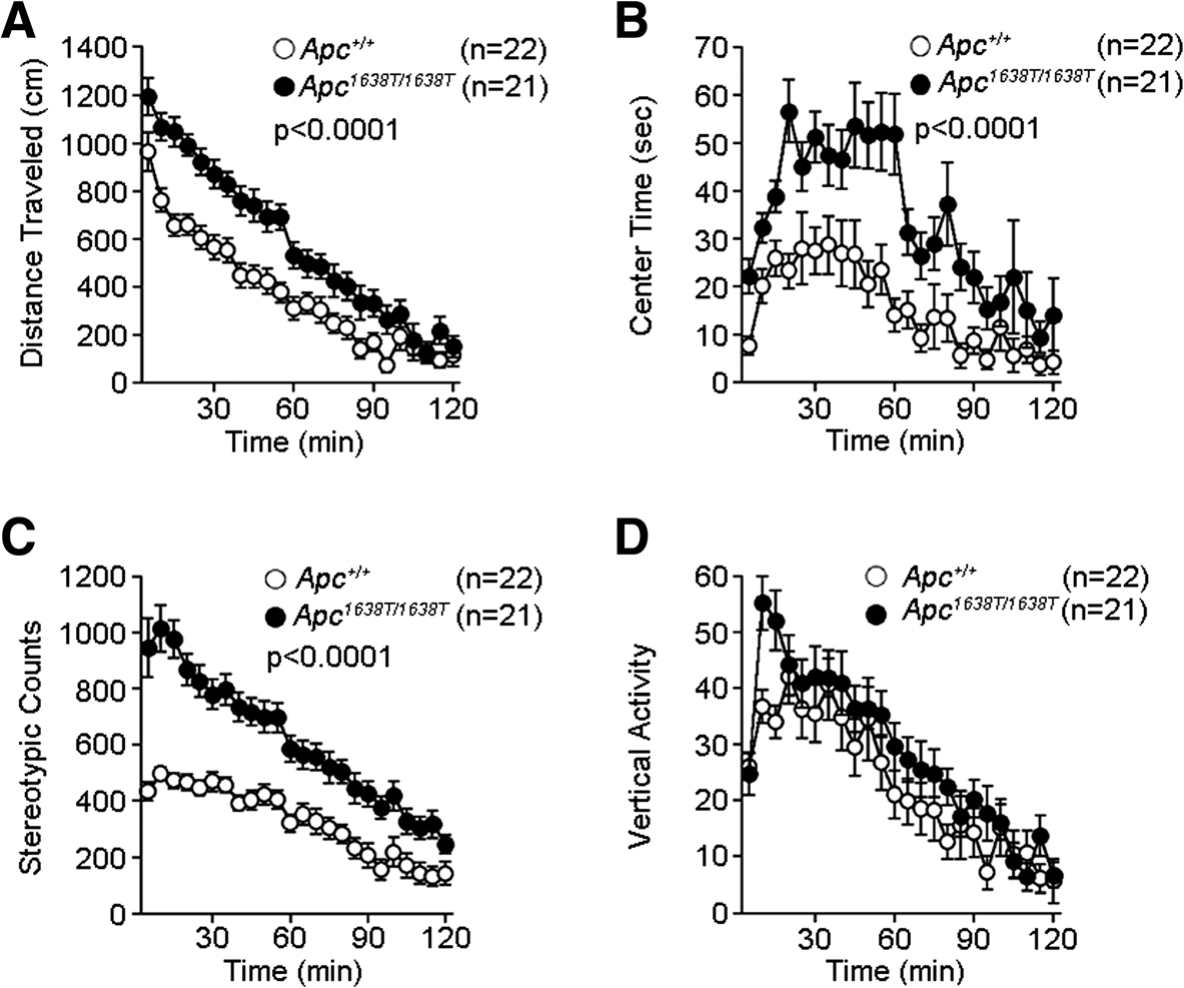
**Increased locomotor activity in open field test in *Apc***^***1638T/1638T ***^**mice. (A)** Distances traveled (genotype effect, F_1,41_ = 22.58, p < 0.0001; genotype × time interaction, F_23,943_ = 2.909, p < 0.0001), **(B)** time spent in the center of the compartment (genotype effect, F_1,41_ = 18.699, p < 0.0001; genotype × time interaction, F_23,943_ = 1.664, p = 0.0258), **(C)** counts of stereotypic behavior (genotype effect, F_1,41_ = 41.58, p < 0.0001; genotype × time interaction, F_23,943_ = 5.233, p < 0.0001), and **(D)** counts of vertical activity were recorded. The p values in the figure indicate genotype effect in two-way repeated measures ANOVA. Error bars indicate SEM.

**Figure 2:**
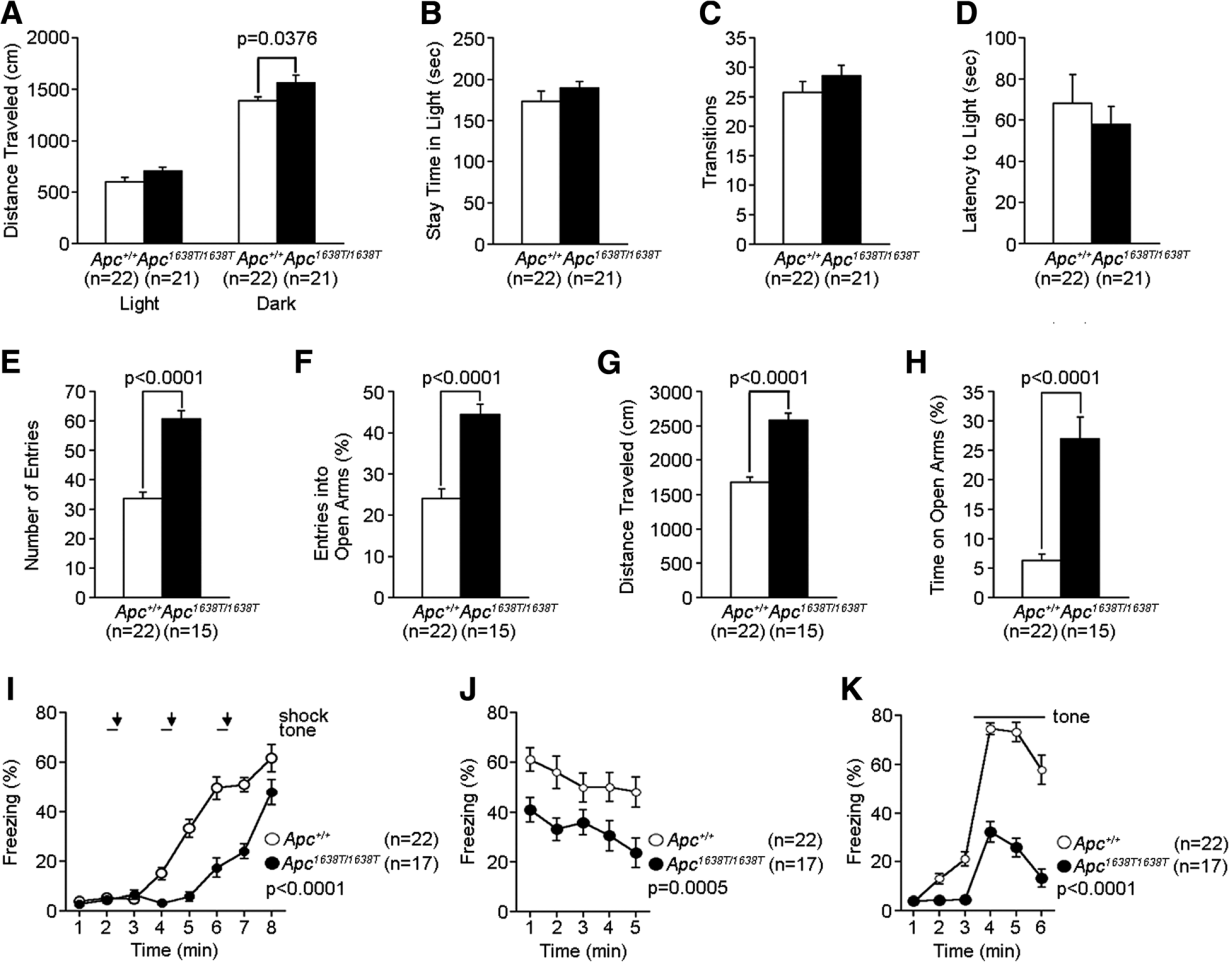
**Decreased anxiety-like behaviors in *Apc***^***1638T/1638T ***^**mice. (A-D)** Light/dark transition test: **(A)** distance traveled in the light/dark compartments, **(B)** time stayed in the light compartment, **(C)** number of light/dark transitions, and **(D)** latency to enter the light compartment were recorded. **(E-H)** Elevated plus maze: **(E)** number of arm entries, **(F)** percentage of entries into the open arms, **(G)** distance traveled, and **(H)** percentage of time spent on open arms were recorded. **(I-K)** Contextual and cued fear conditioning test: **(I)** percentage of time freezing during conditioning (genotype effect, F_1,37_ = 47.089, p < 0.0001; genotype × time interaction, F_7,259_ = 10.618, p < 0.0001), **(J)** percentage of time freezing during contextual testing (genotype effect, F_1,37_ = 14.438, p = 0.0005), and **(K)** percentage of time freezing during cued testing were recorded (genotype effect, F_1,37_ = 75.104, p < 0.0001). During the time indicated by the horizontal bold lines, tone stimuli were given. Arrows indicate the time points of foot shock stimuli. The p values in the figure indicate genotype effect in one-way ANOVA (A-H) or two-way repeated measures ANOVA (I-K). Error bars indicate SEM.

**Figure 3:**
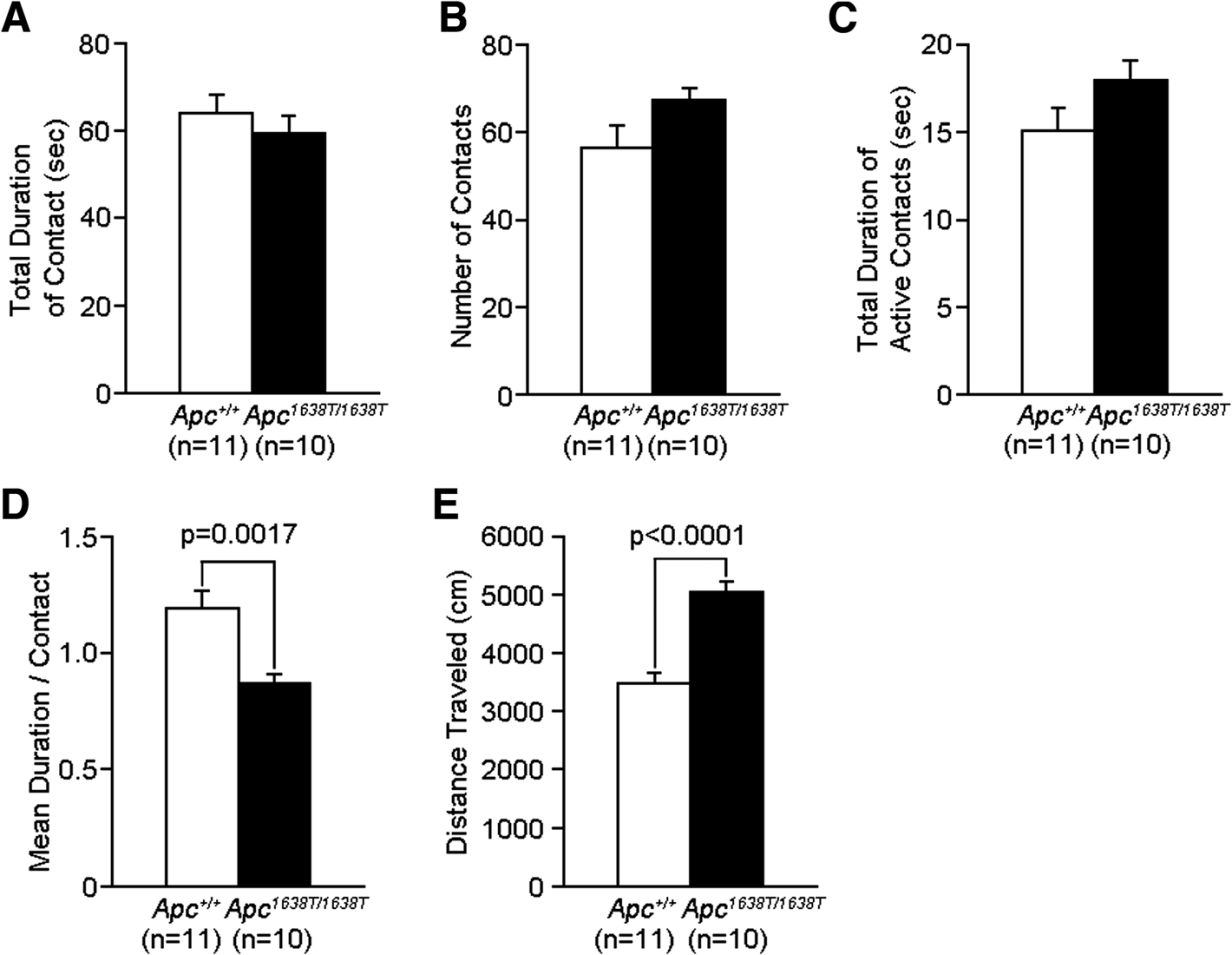
**Mildly decreased social interaction behaviors in a novel environment in *Apc***^***1638T/1638T ***^**mice. (A)** Total duration of contacts, **(B)** number of contacts, **(C)** total duration of active contacts, **(D)** mean duration of each contact, and **(E)** total distance traveled were recorded. The p values indicate genotype effect in one-way ANOVA. Error bars indicate SEM.

**Figure 4:**
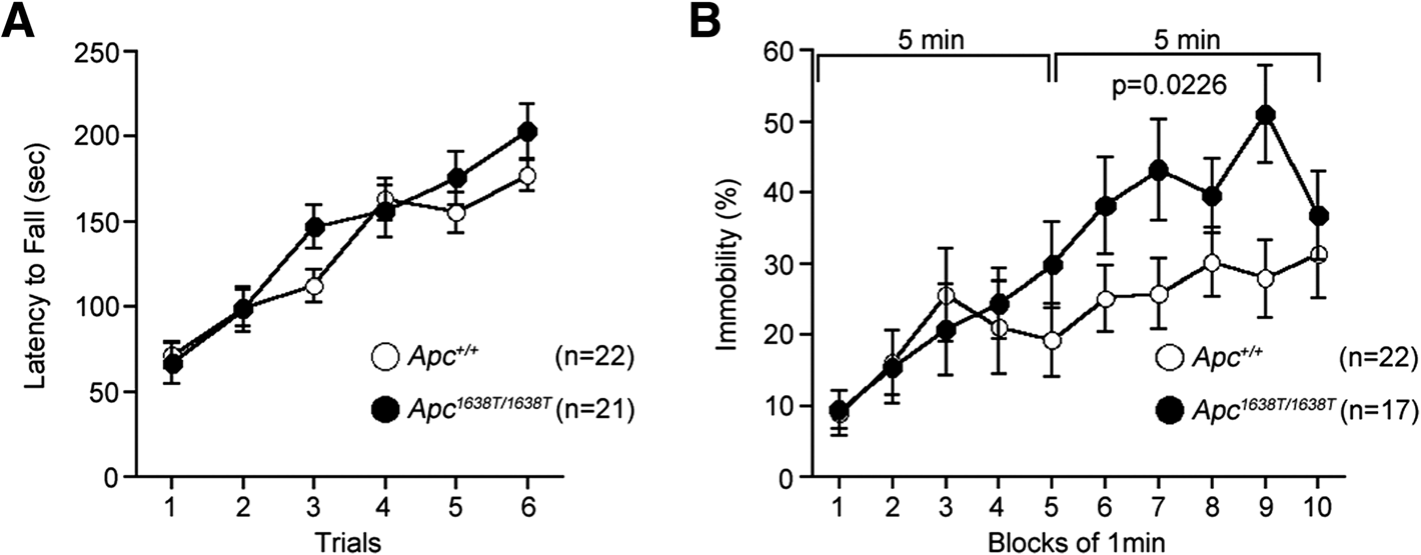
**Mildly increased depression-like behaviors in *Apc***^***1638T/1638T ***^**mice. (A)** Rotarod test: latencies to fall from the rotating drum across training sessions were recorded. **(B)** Tail suspension test: immobility time is presented as the percentage of time in each block (5–10 min, genotype effect, F_1,37_ = 5.663, p = 0.0226). The p value in the figure indicates genotype effect in two-way repeated measures ANOVA. Error bars indicate SEM.

**Figure 5:**
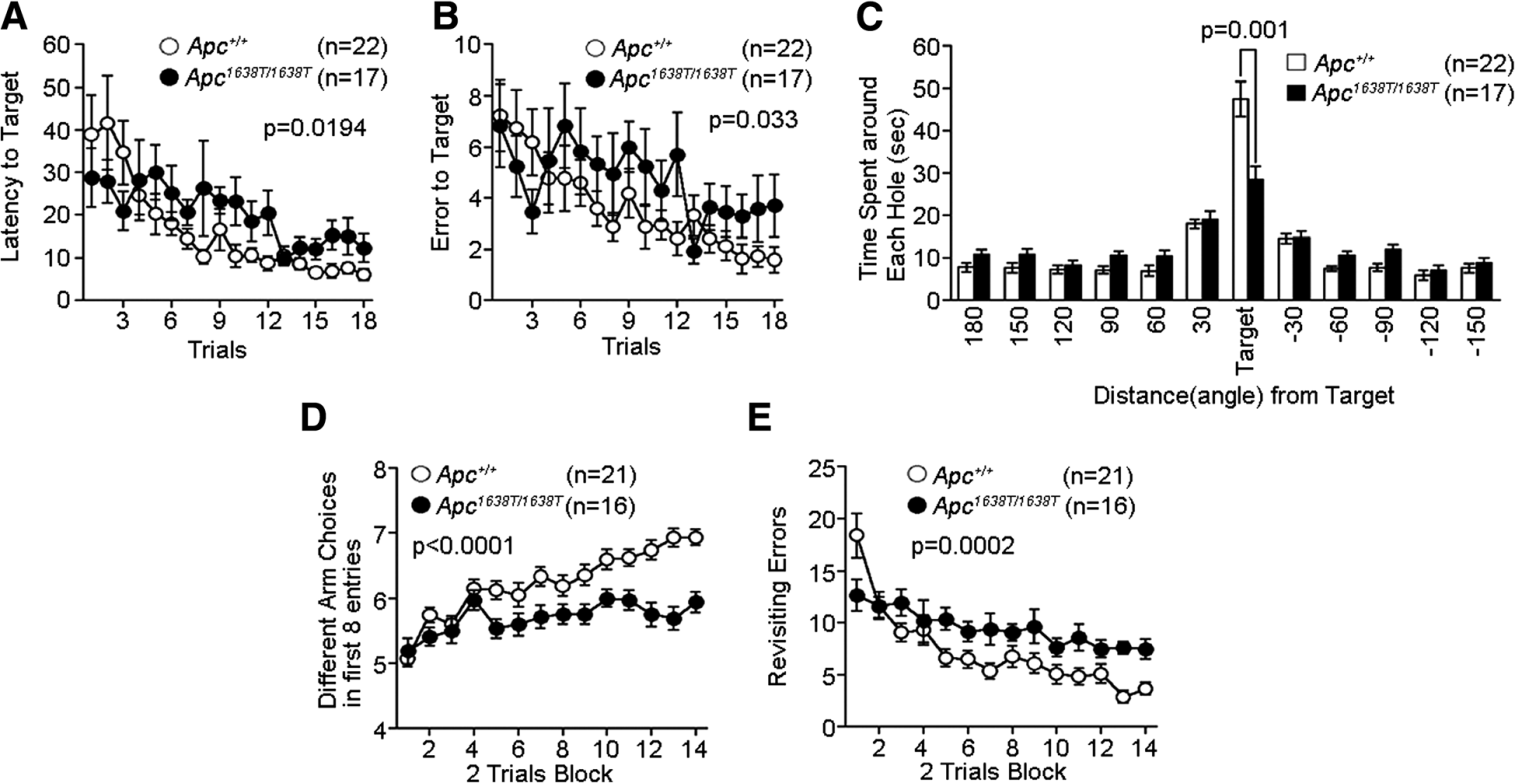
**Impaired learning and memory in *Apc***^***1638T/1638T ***^**mice. (A-C)** Barnes circular maze test: **(A)** latency to reach the target hole (genotype effect, F_1,37_ = 5.974, p = 0.0194) and **(B)** number of errors before reaching the target hole across training sessions (genotype effect, F_1,37_ = 4.904, p = 0.033) were recorded. **(C)** Time spent around each hole in the probe trial conducted 24 hours after the last training session was recorded. **(D and E)** Eight-arm radial maze test: **(D)** different arm choices among the first eight entries (genotype effect, F_1,35_ = 36.684, p < 0.0001; genotype × block of trials interaction, F_16,560_ = 3.109, p < 0.0001), and **(E)** total number of arms revisited during training (genotype effect, F_1,35_ = 16.947, p = 0.0002; genotype × block of trials interaction, F_16,560_ = 2.768, p = 0.0003) were recorded. The p values in the figure indicate genotype effect in two-way repeated measures ANOVA (A, B, D, E) or one-way ANOVA (C). Error bars indicate SEM.

To evaluate the neuropsychological conditions of *Apc*^1638T/1638T^ mice, we conducted a series of behavioral tests. *Apc*^1638T/1638T^ mice appeared healthy, but showed significant changes in some of physical characteristics, including a decrease in body weight (*Apc*^*+/+*^, 26.3 ± 0.2 g; *Apc*^1638T/1638T^, 21.8 ± 0.4 g, p < 0.0001), reduced grip strength (*Apc*^*+/+*^, 0.695 ± 0.033 N; *Apc*^1638T/1638T^, 0.48 ± 0.014 N, p < 0.0001), and impaired wire hang (*Apc*^*+/+*^, > 60 s; *Apc*^1638T/1638T^, 8.2 ± 1.4 s, p < 0.0001). The vision of *Apc*^1638T/1638T^ mice was evaluated by the forepaw reaching test, and all mice showed normal reaching behavior, suggesting that there was no obvious visual defect in the mutants. In the hot plate test, there was no significant difference in the latency to the first hind-paw response between genotypes (Additional file 1: Figure S1), suggesting normal pain sensitivity in the mutants. We performed the open field test to examine locomotor activity (Figure 1). The total distance traveled by *Apc*^1638T/1638T^ mice was significantly greater compared with *Apc*^*+/+*^ mice (Figure 1A), indicating increased locomotor activity in the *Apc*^1638T/1638T^ mice. Hyperactivity was observed in other behavioral tests (see Figure 2A, E and G, and Figure 3E). The mutant mice also showed an increased stereotypic behavior in the open field test (Figure 1C). There was no significant difference between genotypes in the rotarod test (Figure 4A). Next, we performed the Barnes circular maze test to assess spatial reference memory (Figure 5A-C). The latency to find the target hole for *Apc*^1638T/1638T^ mice was significantly longer than for *Apc*^*+/+*^ mice (Figure 5A). Also, the *Apc*^1638T/1638T^ mice made more search errors during acquisition than *Apc*^*+/+*^ mice (Figure 5B). The probe test in which the escape box was removed was performed 24 hours after the last day of the training. In this test, *Apc*^1638T/1638T^ mice stayed near the target for a significantly shorter period of time (Figure 5C). To assess spatial working memory, we performed the eight-arm radial maze test. The number of different arm choices among the first eight entries was significantly lower for *Apc*^1638T/1638T^ mice compared with *Apc*^*+/+*^ mice (Figure 5D), and the number of revisiting errors was significantly higher in *Apc*^1638T/1638T^ mice (Figure 5E). These results suggest that *Apc*^1638T/1638T^ mice have a deficit in spatial reference and working memory.

In addition to altered spatial learning and memory, *Apc*^1638T/1638T^ mice showed mildly increased depression-like behavior in the tail suspension test (Figure 4B), decreased anxiety-like behavior in the open field (Figure 1B) and elevated plus maze tests (Figure 2F and H), but not in the light/dark transition test (Figure 2A-D), and mildly decreased social interaction behavior (Figure 3D). *Apc*^1638T/1638T^ mice demonstrated decreased freezing in the fear conditioning test in both conditioning and testing phases (Figure 2I-K). Although the result of the hot plate test suggested intact pain sensitivity in *Apc*^1638T/1638T^ mice (see above), decreased freezing during the conditioning phase in the mutant mice may be due to reduced sensitivity to the electrical footshock. To examine shock sensitivity, we measured distance traveled during the footshocks (Additional file 2: Figure S2). *Apc*^1638T/1638T^ mice and wild-type mice similarly responded to the first footshock, but *Apc*^1638T/1638T^ mice traveled a shorter distance in response to the second and third footshocks. Therefore, while *Apc*^1638T/1638T^ mice appear to have normal pain sensitivity, adaptation to pain or fear during repeated delivery of footshocks could be enhanced in the mutant mice. In the startle response and prepulse inhibition tests, there were no consistent differences between genotypes (Additional file 3: Figure S3). Taken together, these results suggest that the deletion of the C-terminus of Apc causes robust changes in cognitive and neuropsychiatric functions.

### Altered synapse morphology and function in *Apc*^*1638T/1638T*^ mice

**Figure 6:**
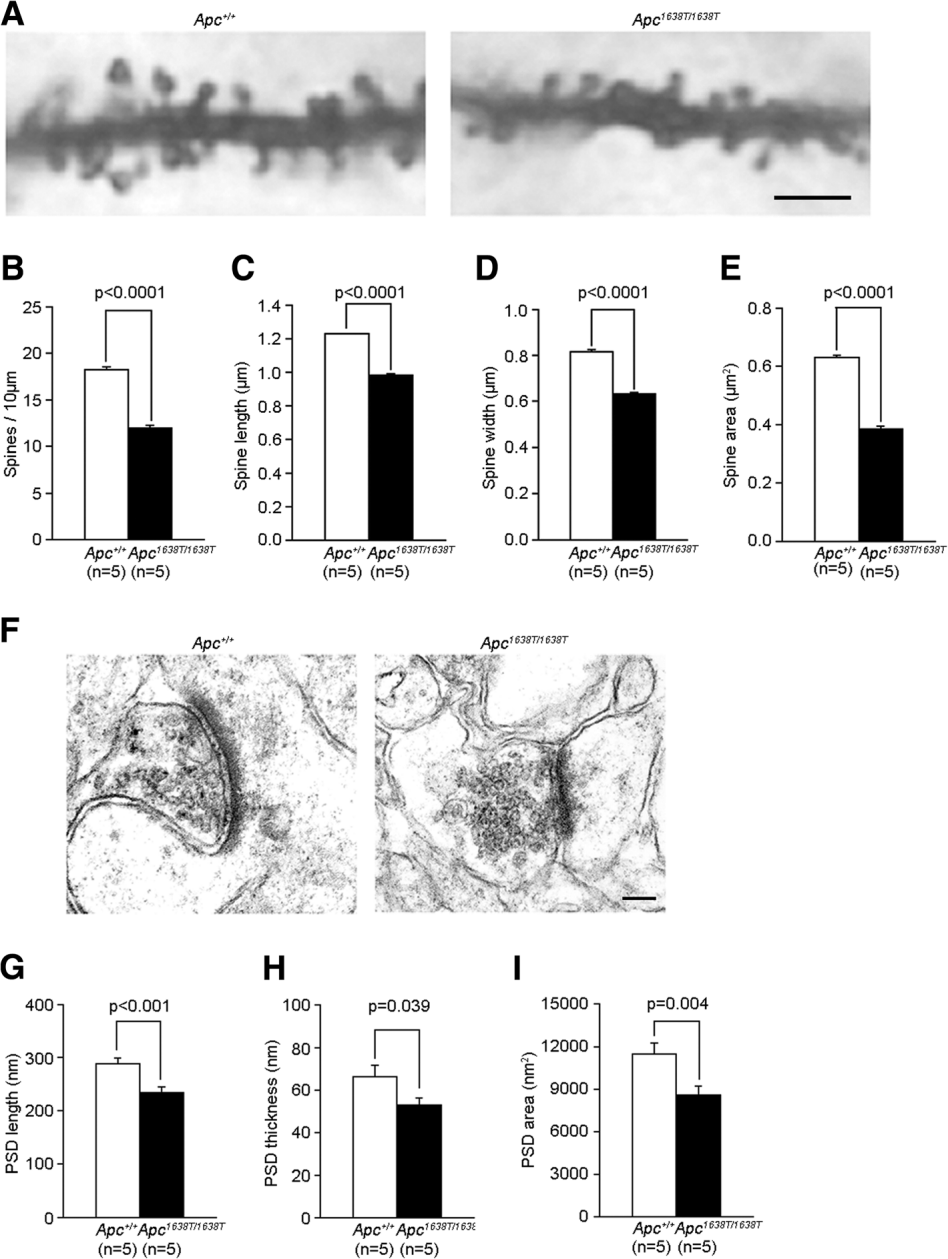
**Altered synapse morphology in *Apc***^***1638T/1638T ***^**mice. (A)** Golgi impregnated dendrites in the stratum oriens of the hippocampal CA1 region. The scale bar represents 2 μm. **(B-E)** Quantification of dendritic spine: **(B)** density, **(C)** length, **(D)** width, and **(E)** area. **(F)** Representative electron photomicrographs of hippocampal synapses. The PSD is visible as an electron-dense layer adjacent to the postsynaptic membrane. The scale bar represents 0.1 μm. **(G-I)** Quantification of PSD: **(G)** length, **(H)** thickness, and **(I)** area. Two-tailed Student’s t-test was used for statistical analysis.

The hippocampal CA1 region is a well-known crucial site for processing associative memories that typically contain information about what, where, and when major events occurred [11]. In order to investigate the potential cellular correlate of the behavioral phenotypes, we performed morphological and electrophysiological analyses of the hippocampal CA1 region in the *Apc*^1638T/1638T^ mice. While no differences were found in the number of neurons in the CA1 region between *Apc*^1638T/1638T^ and *Apc*^*+/+*^ mice (Additional file 4: Figure S4A-S4C), the dendritic spine density in *Apc*^1638T/1638T^ mice was reduced compared with *Apc*^*+/+*^ mice (Figure 6A-B). The size of the spines was also smaller in *Apc*^1638T/1638T^ mice (Figure 6C-E). The postsynaptic density (PSD) of the postsynaptic spines in *Apc*^1638T/1638T^ mice was also smaller in length, thickness, and area than those in *Apc*^*+/+*^ mice (Figure 6F-I).

**Figure 7:**
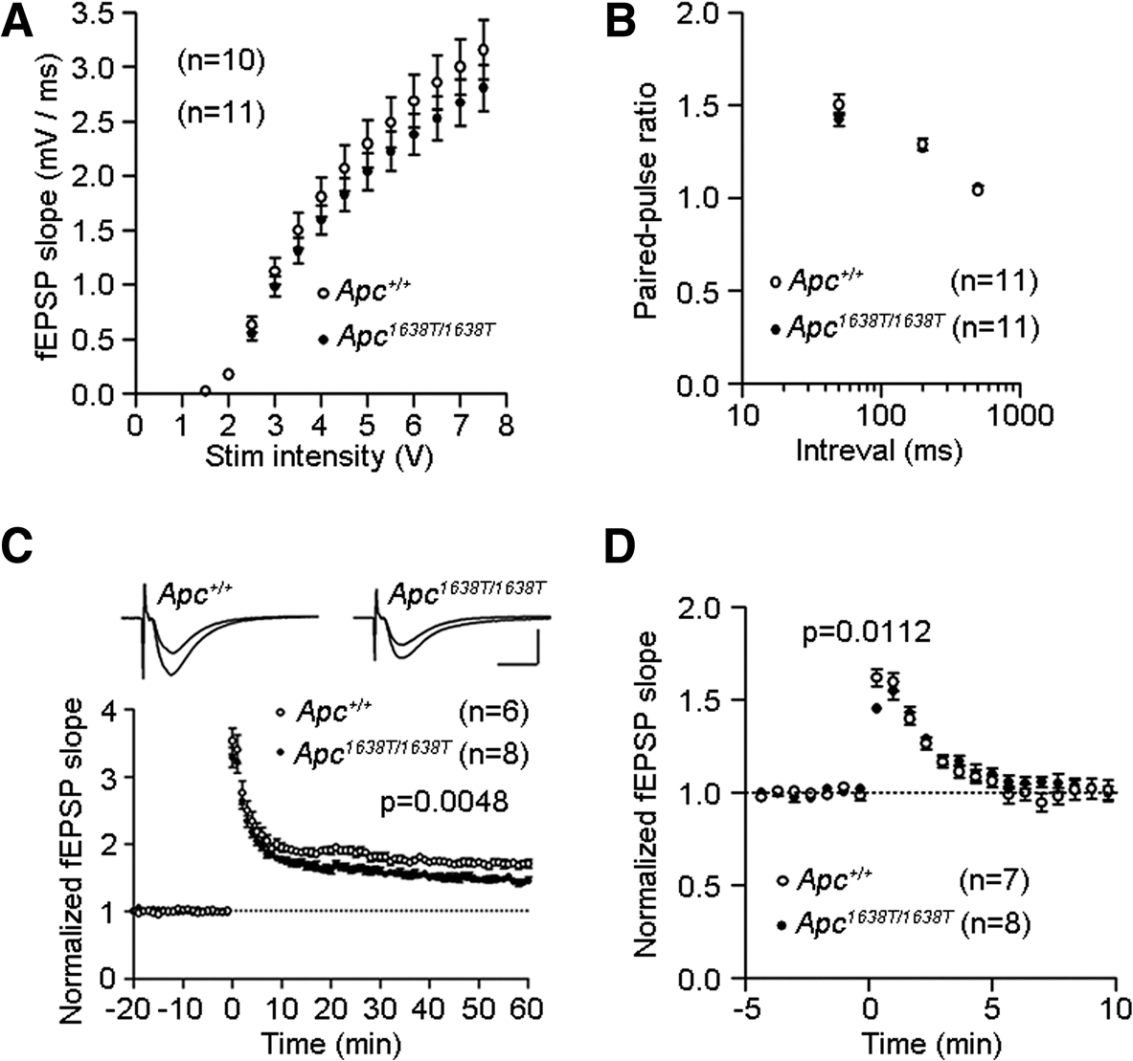
**Altered excitatory synaptic transmission in the hippocampal CA1 region of *Apc***^***1638T/1638T ***^**mice. (A)** Input–output relationship and **(B)** paired-pulse facilitation. **(C)** Long-term potentiation induced in standard saline. Tetanic stimulation was applied at 0 min. Scale bars, 10 ms, 0.5 mV. **(D)** Post-tetanic potentiation in the presence of D-APV. The number of slices used is indicated by “n”. Two-tailed Student’s t-test was used for statistical analysis.

Although abnormalities in the structure of spines and synapses were observed in the hippocampal CA1 region of *Apc*^1638T/1638T^ mice, the basic properties of hippocampal excitatory synaptic transmission were generally normal (Figure 7A and B). However, the magnitude of long-term potentiation (LTP) was reduced at the Schaffer collateral/commissural fiber-CA1 synapses in *Apc*^1638T/1638T^ mice (Figure 7C and Additional file 5: Figure S5). In addition, the initial peak of post-tetanic potentiation (PTP) immediately after the tetanic stimulation was reduced in *Apc*^*1638T/1638T*^ mice with no significant change in its decay to the baseline (Figure 7D). These results indicate impairment of activity-dependent synaptic modifications in the hippocampal CA1 neurons of *Apc*^1638T/1638T^ mice.

### Altered amounts of dopamine and serotonin in various brain regions of *Apc*^*1638T/1638T*^ mice

**Figure 8:**
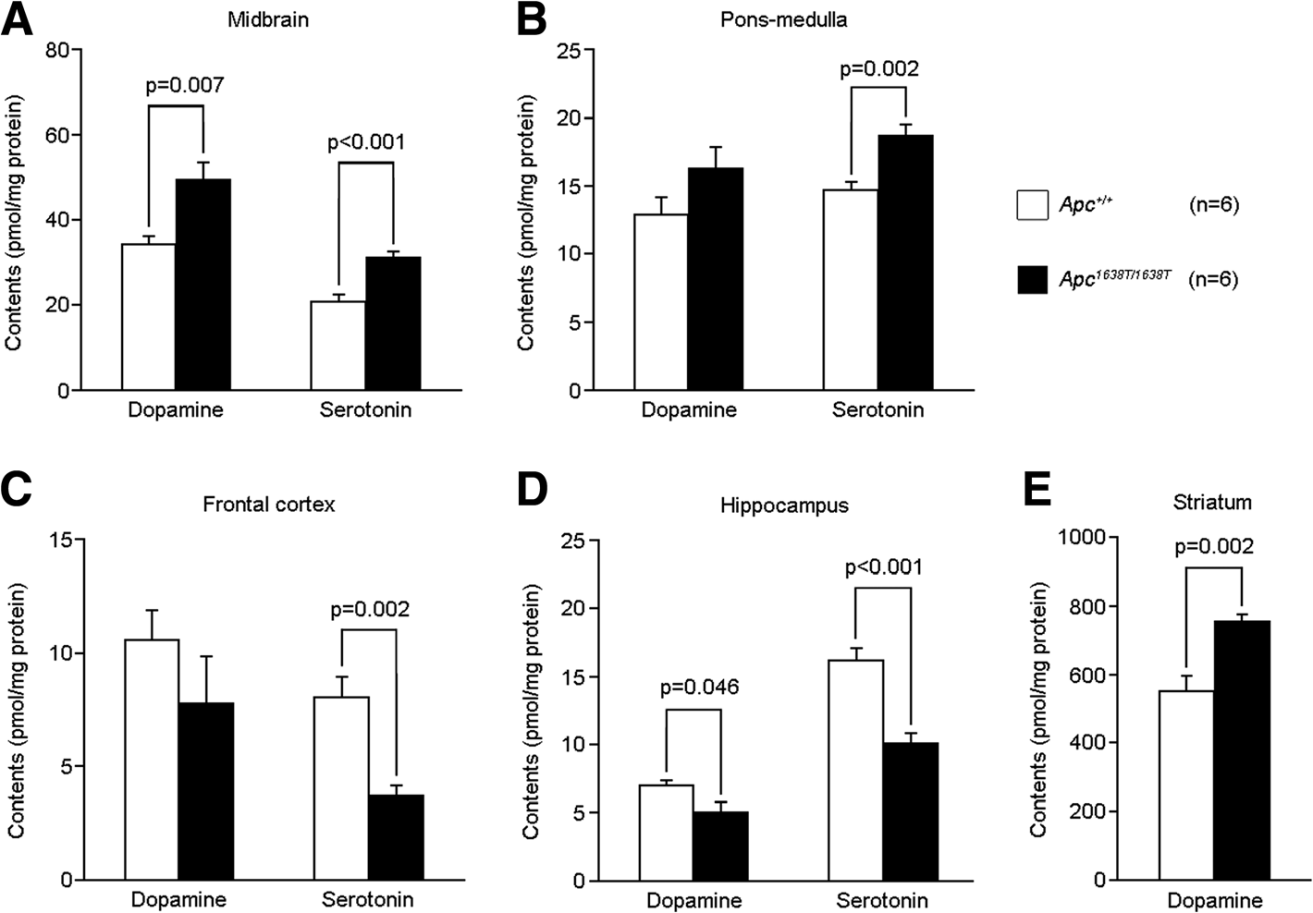
**Altered amounts of dopamine and serotonin in various brain regions of *Apc***^***1638T/1638T ***^**mice.** Amounts of dopamine and serotonin in **(A)** midbrain, **(B)** pons-medulla, **(C)** frontal cortex, **(D)** hippocampus, and **(E)** striatum. Error bars indicate SEM. Two-tailed Student’s t-test was used for statistical analysis.

Alterations in monoamine neurotransmission are often associated with schizophrenia and mood disorders, and the activities of glutamatergic neurons in the prefrontal cortex and mesencephalic dopaminergic neurons are mutually regulated by GABAergic interneurons [12]. We analyzed the amounts of monoamines and metabolites in selected brain regions. In *Apc*^1638T/1638T^ mice, the amounts of dopamine and serotonin in the midbrain and pons-medulla, where catecholaminergic neuronal cell bodies are located, were significantly increased (Figure 8A and B). In contrast, the amounts of dopamine and serotonin in the hippocampus, and of serotonin in the frontal cortex, where catecholaminergic nerve fibers terminals terminate, were decreased (Figure 8C and D). There were higher amounts of dopamine in the striatum of *Apc*^1638T/1638T^ mice than in *Apc*^*+/+*^ mice (Figure 8E). Also, significant alterations in the amounts of norepinephrine, dihydroxyphenylacetic acid, homovanillic acid, and 5-hydroxyindolacetic acid were found in the brain regions assayed (Table 1). The altered distribution of the monoamines and their metabolites in *Apc*^1638T/1638T^ mice was similar to what has been observed in some animal models of schizophrenia, such as phencyclidine-treated mice and heparin-binding epidermal growth factor-like growth factor-deficient mice [13,14].

**Table 1:** **Abnormal amounts of monoamines and their metabolites in the brain of *Apc***^***1638T/1638T ***^**mice**

**Brain region**	**Genotype**		**Norepinephrine**	**DOPAC**	**HVA**	**5-HIAA**
			**pmol/mg protein**			
Midbrain	*Apc*^*+/+*^	(n = 6)	13.4 ± 0.8	5.46 ± 0.42	9.21 ± 0.93	58.8 ± 2.6
	*Apc*^*1638T/1638T*^	(n = 6)	29.5 ± 1.8****	7.57 ± 0.65*	11.4 ± 1.19	78.7 ± 4.9***
Pons-medulla	*Apc*^*+/+*^	(n = 6)	10.7 ± 0.6	1.34 ± 0.15	2.13 ± 0.36	29.4 ± 1.5
	*Apc*^*1638T/1638T*^	(n = 6)	23.9 ± 1.1****	2.13 ± 0.16***	2.77 ± 0.33	37.2 ± 1.9**
Striatum	*Apc*^*+/+*^	(n = 6)	nd	113 ± 6.0	76.9 ± 3.9	20.9 ± 0.9
	*Apc*^*1638T/1638T*^	(n = 6)	nd	173 ± 7.6****	94.3 ± 5.5*	24.7 ± 1.9
Frontal cortex	*Apc*^*+/+*^	(n = 6)	8.48 ± 0.69	2.74 ± 0.75	5.16 ± 0.87	11.8 ± 0.5
	*Apc*^*1638T/1638T*^	(n = 6)	2.62 ± 0.34****	3.53 ± 0.64	6.04 ± 0.70	9.78 ± 0.76*
Hippocampus	*Apc*^*+/+*^	(n = 6)	10.1 ± 0.6	1.21 ± 0.18	3.11 ± 0.24	27.9 ± 3.0
	*Apc*^*1638T/1638T*^	(n = 6)	2.42 ± 0.29****	1.52 ± 0.16	4.09 ± 0.40	22.1 ± 1.5

Amounts of monoamines and their metabolites are expressed as pmol/mg protein. Values are the means ± SEM. nd: not detected, DOPAC: dihydroxyphenylacetic acid, HVA: homovanillic acid, 5-HIAA: 5- hydroxyindolacetic acid. *p < 0.05, **p < 0.01, ***p < 0.005, ****p < 0.001.

## Discussion

In the present study, our comprehensive behavioral test battery revealed that *Apc*^1638T/1638T^ mice shows impaired learning and memory, increased locomotor activity, mildly increased depression-like behavior, reduced anxiety-like behavior and mildly decreased social interaction behavior. In the hippocampal CA1 region of *Apc*^1638T/1638T^ mice, the dendritic spine density and size are reduced, the PSD was smaller, and LTP was impaired. Taken together, our findings provide the first direct evidence of neuropsychological roles for the C-terminus of Apc tumor suppressor.

*Apc*^1638T/1638T^ mice showed hyperactivity, impaired memory, increased depression-like behavior, and decreased social interaction. These behavioral characteristics are often linked to symptoms of schizophrenia (see Supplementary Table 11 in [15]) and observed in many animal models of schizophrenia [15-19]. In particular, *Apc*^1638T/1638T^ mice showed a marked deficit in the performance of the working memory task. Impaired working memory is commonly observed in patients with schizophrenia and is thought to be a cognitive endophenotype of schizophrenia [20]. On the other hand, the mutant mice did not show clear deficits in prepulse inhibition, another endophenotype of schizophrenia. We also found a marked reduction in the density and size of dendritic spines in the hippocampal CA1 region of *Apc*^1638T/1638T^ mice. A reduction in spine density has been reported in patients with schizophrenia [21], and a reduction in hippocampal spine density and size has also been reported in various animal models of schizophrenia [13,17,22,23]. Therefore, the behavioral and neuronal phenotypes of *Apc*^1638T/1638T^ mice are related to some of symptoms and endophenotypes or neuropathology of schizophrenia.

Synapses have been proposed to be the major site responsible for the pathogenesis of schizophrenia [24]. In addition to changes in the spine density and size, we found marked alterations in the morphology of synapses in the hippocampal CA1 region of *Apc*^1638T/1638T^ mice. The PSD of the postsynaptic spines in the hippocampus of *Apc*^1638T/1638T^ mice was smaller in length, thickness, and area than those in *Apc*^*+/+*^ mice. Association between morphological changes in the dendritic spines and impairment of LTP has been reported [25]. Consistently, we found reduced LTP in the CA1 region of *Apc*^1638T/1638T^ mice. Physiological, pharmacological, and genetic interventions that alter or occlude LTP are accompanied by impairments in memory maintenance [26]. Therefore, it is possible that the changes in the hippocampal spines, synaptic morphology and synaptic function in *Apc*^1638T/1638T^ mice could be a cellular correlate of impaired reference and/or working memory in these mice. Anatomical and functional abnormalities in the hippocampus have been consistently demonstrated in schizophrenia [27]. Patients with schizophrenia show altered working memory-related hippocampal activity [28-30]. Our present findings in mice support these previous observations in humans. However, the relatively mild impairment of synaptic plasticity observed in the CA1 region suggests a contribution of dysfunction of other brain regions to severe memory deficits in *Apc*^1638T/1638T^ mice. The prefrontal cortex has been implicated in working memory deficits in schizophrenia [31]. Since Apc is widely expressed in the neocortex [2], the *Apc*^1638T^ mutation may cause similar impairment of synaptic transmission in such brain region.

Dysfunction of glutamatergic synapses, especially N-methyl-D-aspartate (NMDA) receptor hypofunction, has been implicated in schizophrenia [32]. PSD-95 is a major glutamatergic membrane-associated guanylate kinase protein with three PDZ domains. APC selectively binds the second PDZ domain of PSD-95 through its C-terminal sequence [4]. APC and PSD-95 have been suggested to be involved in the clustering of glutamate NMDA receptors [4,33] and α-Amino-3-hydroxy-5-methyl-4-isoxazolepropionic acid (AMPA) receptors [34] at the postsynaptic membrane. The truncated Apc1638T protein cannot interact with PSD-95 as it does not comprise the corresponding binding motif [10] and therefore may cause a decrease in synaptic accumulation of AMPA and NMDA receptors. The smaller PSD in *Apc*^1638T/1638T^ mice also suggests possible impairment of synaptic expression of AMPA and NMDA receptors, because these receptors are embedded in the PSD. However, our electrophysiological analysis showed no significant changes in basal properties of the excitatory synaptic transmission in the CA1 region of *Apc*^1638T/1638T^ mice, suggesting intact synaptic expression of AMPA receptors in the mutant mice. The attenuation of LTP in the mutant mice suggests that NMDA receptor-dependent signaling activated by high-frequency stimulation is impaired, which is consistent with the above prediction. Since the initial peak of PTP measured in the presence of the NMDA receptor antagonist was also reduced, it is possible that presynaptic functions or short-lasting presynaptic modulations activated by high-frequency stimulation are altered in the mutant mice.

A recent study showed an age-dependent working memory deficit in *Apc* heterozygous knockout (*Apc*^+/-^) mice [9]. Although possible effects of tumors on the performance of behavioral tasks cannot be excluded in *Apc*^+/-^ mice, this result is consistent with our present finding in *Apc*^1638T/1638T^ mice. In contrast to the hyperactive phenotype in *Apc*^1638T/1638T^ mice, however, *Apc*^+/-^ mice exhibited hypoactivity in various behavioral tests. In addition, *Apc*^+/-^ mice showed no significant changes in anxiety-related behaviors or social interaction in the same behavioral tests as used in the present study. These results support a critical importance of the C-terminus of Apc in regulating neuropsychiatric behaviors. The hypoactive phenotype of *Apc*^+/-^ mice may be ascribed to impairment of Wnt/β-catenin signaling.

## Conclusions

The C-terminus of the Apc tumor suppressor plays a critical role in cognitive and neuropsychiatric functioning. Our behavioral, morphological, electrophysiological, and biochemical analyses revealed the neurological phenotypes of *Apc*^1638T/1638T^ mice, many of which are related to the symptom and endophenotype of schizophrenia. Our finding is consistent with the previous report of the association between *APC* and susceptibility for schizophrenia [7]. As the targeted mutation in these animals selectively deletes the C-terminus of Apc, the observed phenotype is unlikely attributable to altered Wnt signaling and may be due to a failure of PSD-95 to interact with Apc. More in-depth molecular and neurobiological analyses of *Apc*^1638T/1638T^ mice may provide novel therapeutic strategies for the treatment of schizophrenia and related psychiatric disorders.

## Methods

### Animals and experimental design

For the present analyses, *Apc*^*1638T/1638T*^ mice [10] were backcrossed to C57BL/6JJmsSlc by 10 times to increase litter size. The resulting heterozygotes were then intercrossed to produce homozygous *Apc*^*1638T/1638T*^ mice. *Apc*^*+/+*^ littermates were used as controls.

For behavioral analyses, mice were group-housed (4 mice per cage) in a room with a 12 hours light/dark cycle (lights on at 7:00 a.m.) with access to food and water ad libitum unless otherwise stated. Behavioral testing was performed between 9:00 a.m. and 6:00 p.m., excepting for that of eight-arm radial maze and Barnes circular maze that were performed between 8:00 p.m. and 5:00 a.m.. After the tests, all apparatus were cleaned with diluted hypochlorite solution to prevent a bias due to olfactory cues. All behavioral tests were conducted in a manner similar to those previously described [19,35]. All behavioral testing procedures were approved by the Animal Care and Use Committee of Kyoto University Graduate School of Medicine, and by the Animal Research Committee of the National Institute for Physiological Sciences. The raw data of behavioral tests, which are not described in this paper, are disclosed in the gene-brain-phenotyping database (http://www.mouse-phenotype.org/).

For morphological and biochemical analyses, mice were housed one per cage in a room with a 12 hours light/dark cycle (lights on at 8:00 a.m.) with access to food and water ad libitum. All morphological analyzing procedures were approved by the Institutional Animal Care and Use Committee of Fujita Health University.

### Open field test

Locomotor activity was measured using the open field test. Open field test was performed with 10 to 12 weeks old male mice. Each mouse was placed in the center of the open field apparatus (40 × 40 × 30 cm; Accuscan Instruments, Columbus, OH, USA). Total distance traveled (in cm), vertical activity (rearing measured by counting the number of photobeam interruptions), time spent in the center, the beam-break counts for stereotypic behaviors (repeated beam breaks on the same beam, typically caused by grooming or sniffing), and the number of fecal boli were recorded. Data were collected for 120 min. Photobeams and the detectors (x,y,z direction) were spaced 2.5 cm to measure mouse activity. The center area was defined as 20 cm × 20 cm area located at the center of the open field.

### Rotarod test

Motor coordination and balance were tested with the rotarod test. The rotarod test using an accelerating rotarod (UGO Basile Accelerating Rotarod, Comerio, Varese, Italy) was performed by placing 12 to 15 weeks old male mice on rotating drum (3 cm diameter) and measuring the time during which each animal was able to maintain its balance on the rod as latency time to fall (sec). The speed of the rotarod accelerated from 4 to 40 rpm over a 5 min period.

### Barnes circular maze test

Barnes circular maze test was performed with 21 to 27 weeks old male mice. The test was conducted on dry land, a white circular surface, 1.0 m in diameter, with 12 holes equally spaced around the perimeter (O’Hara & Co., Tokyo, Japan). The circular open field was elevated 75 cm from the floor. A black Plexiglas escape box (17 × 13 × 7 cm), which had paper cage bedding on its bottom, was located under one of the holes. The hole above the escape box represented the target, analogous to the hidden platform in the Morris task. The location of the target was consistent for a given mouse, but was randomized across mice. The maze was rotated daily, with the spatial location of the target unchanged with respect to the distal visual room cues, to prevent a bias based on olfactory or proximal cues within the maze. Three trials per day were conducted for 6 successive days. On day 7, a probe trial was conducted without the escape box, to confirm that this spatial task was acquired based on navigation using distal environment room cues. The number of errors to reach the target hole and the time spent around each hole were recorded by video tracking software (Image BM).

### Eight-arm radial maze test

Eight-arm radial maze test was performed with 16 to 22 weeks old male mice. Fully-automated eight-arm radial maze apparatuses (O’Hara & Co., Tokyo, Japan) were used. The floor of the maze was made of white plastic, and the wall (25 cm high) consisted of transparent plastic. Each arm (9 × 40 cm) radiated from an octagonal central starting platform (perimeter 12 × 8 cm) like the spokes of a wheel. Identical food wells (1.4 cm deep and 1.4 cm in diameter) with pellet sensors were placed at the distal end of each arm. The pellets sensors were able to automatically record pellet intake by the mice. The maze was elevated 75 cm above the floor and placed in a dimly-lit room with several extra-maze cues. During the experiment, the maze was maintained in a constant orientation. One week before pretraining, animals were deprived of food until their body weight was reduced to 80% to 85% of the initial level. Pretraining started on the 8th day. Each mouse was placed in the central starting platform and allowed to explore and consume food pellets scattered on the whole maze for a 30 min period (one session per mouse). After completion of the initial pretraining, mice received another pretraining to take a food pellet from each food well after being placed at the distal end of each arm. A trial was finished after the mouse consumed the pellet. This was repeated eight times, using eight different arms, for each mouse. After these pretraining trials, actual maze acquisition trials were performed. In the spatial working memory task of the eight-arm radial maze, all eight arms were baited with food pellets. Mice were placed on the central platform and allowed to obtain all eight pellets within 25 min. A trial was terminated immediately after all eight pellets were consumed or 25 min had elapsed. An arm visit was defined as traveling more than 5 cm from the central platform. The mice were confined at the center platform for 5 sec after each arm choice. The animals went through one trial per day. For each trial, arm choice, latency to obtain all pellets, distance traveled, number of different arms chosen within the first eight choices, the number of arm revisited, and omission errors were automatically recorded. Data acquisition, control of guillotine doors, and data analysis were performed by Image RM software.

### Tail suspension test

Tail suspension test was performed with 26 to 28 weeks old male mice. Mice were suspended 30 cm above the floor in a visually isolated area by adhesive tape placed 1 cm from the tip of the tail, and their behavior was recorded over a 10 min test period. Data acquisition and analysis were performed automatically, using ImageTS software.

### Startle response/prepulse inhibition tests

Startle response/prepulse inhibition tests were performed with 13 to 16 weeks old and 28 to 31 weeks old mice. A startle reflex measurement system was used (O’Hara & Co., Tokyo, Japan) to measure startle response and prepulse inhibition. A test session began by placing a mouse in a plastic cylinder where it was left undisturbed for 10 min. White noise (40 msec) was used as the startle stimulus for all trial types. The startle response was recorded for 140 msec (measuring the response every 1 msec) starting with the onset of the prepulse stimulus. The background noise level in each chamber was 70 dB. The peak startle amplitude recorded during the 140 msec sampling window was used as the dependent variable. A test session consisted of six trial types (i.e., two types for startle stimulus only trials, and four types for prepulse inhibition trials). The intensity of the startle stimulus was 110 or 120 dB. The prepulse sound was presented 100 msec before the startle stimulus, and its intensity was 74 or 78 dB. Four combinations of prepulse and startle stimuli were used (74–110, 78–110, 74–120, and 78–120 dB). Six blocks of the six trial types were presented in pseudorandom order such that each trial type was presented once within a block. The average inter-trial interval was 15 sec (range: 10–20 sec).

### Light/dark transition test

Light/dark transition test was performed with 10 to 13 weeks old male mice. The apparatus used for light/dark transition test consisted of a cage (21 × 42 × 25 cm) divided into two sections of equal size by a partition containing a door (O’Hara & Co., Tokyo, Japan). One chamber was brightly illuminated (390 lux), whereas the other chamber was dark (2 lux). Mice were placed into the dark side, and allowed to move freely between the two chambers with the door open for 10 min. The total number of transitions between chambers, time spent in each compartment, first latency to enter the light side and distance traveled were recorded automatically using ImageLD software.

### Elevated plus-maze test

Elevated plus-maze test was performed with 11 to 13 weeks old male mice. The elevated plus-maze (O’Hara & Co., Tokyo, Japan) consisted of two open arms (25 × 5 cm) and two enclosed arms of the same size, with 15 cm high transparent walls. The arms and central square were made of white plastic plates and were elevated to a height of 55 cm above the floor. In order to minimize the likelihood of animals falling from the apparatus, 3 mm high plastic ledges were provided for the open arms. Arms of the same type were arranged at opposite sides to each other. Each mouse was placed in the central square of the maze (5 × 5 cm), facing one of the closed arms. Mouse behavior was recorded during a 10 min test period. The number of entries into, and the time spent in open and enclosed arms were recorded. For data analysis, we used the following four measures: the percentage of entries into the open arms, the time spent in the open arms (sec), the number of total entries, and total distance traveled (cm). Animals that fell down from the apparatus during the recording were excluded from the analysis. Data acquisition and analysis were performed automatically, using ImageEP software.

### Contextual and cued fear conditioning tests

Contextual and cued fear conditioning tests were performed with 26 to 29 weeks old male mice. Each mouse was placed in a test chamber (26 × 34 × 29 cm) inside a sound-attenuated chamber (O’Hara & Co., Tokyo, Japan) and allowed to explore freely for 2 min. A 60 dB white noise, which served as the conditioned stimulus (CS), was presented for 30 sec, followed by a mild (2 sec, 0.35 mA) foot shock, which served as the unconditioned stimulus (US). Two more CS–US pairings were presented with 2 min inter-stimulus interval. To examine shock sensitivity, we measured distance traveled when the foot shock were delivered (from 2 sec before the shock to 2 sec after the shock, total 6 sec). Context testing was conducted 1 day after conditioning in the same chamber. Cued testing with altered context was conducted 1 day after conditioning using a triangular box (35 × 35 × 40 cm) made of white opaque plastic, which was located in a different room. Data acquisition, control of stimuli (i.e., tones and shocks), and data analysis were performed automatically, using ImageFZ software. Images were captured at one frame per second. For each pair of successive frames, the amount of area (pixels) by which the mouse moved was measured. When this area was below a certain threshold (i.e., 20 pixels), the behavior was judged as freezing. When the amount of area equaled or exceeded the threshold, the behavior was considered as non-freezing. The optimal threshold (amount of pixels) to judge freezing was determined by adjusting it to the amount of freezing measured by human observation. Freezing that lasted less than the defined time threshold (i.e., 2 sec) was not included in the analysis.

### Social interaction test in a novel environment

In the social interaction test was performed with 11 to 14 weeks old male mice. Two mice of identical genotypes that were previously housed in different cages were placed in a box together (35 × 35 × 40 cm) and allowed to explore freely for 10 min. Social behavior was monitored with a CCD camera connected to a Macintosh computer. Analysis was performed automatically using ImageSI software. The total number of contacts, total duration of active contacts, total contact duration, mean duration per contact, and total distance traveled were measured. The active contact was defined as follows. Images were captured at 1 frame per second, and distance traveled between two successive frames was calculated for each mouse. If the two mice contacted each other and the distance traveled by either mouse was longer than 2 cm, the behavior was considered as an active contact.

### Image analysis

The applications used for the behavioral studies (ImageLD, ImageEP, ImageTS, ImageFZ, ImageRM, ImageBM, and ImageSI) were based on the public domain NIH Image program (developed at the U.S. National Institutes of Health and available on the Internet at http://rsb.info.nih.gov/nih-image/) and ImageJ program (http://rsb.info.nih.gov/ij/), which were modified for each test by Tsuyoshi Miyakawa (available through O’Hara & Co., Tokyo, Japan). ImageLD, ImageEP, and ImageFZ are freely available from http://www.mouse-phenotype.org/software.html.

### Immunofluorescence

For histological analyses in hippocampal CA1, immunofluorescence labeling for neuron-specific nuclear protein (NeuN) was performed. Mice (10 weeks old, male) were perfused with 4% paraformaldehyde (PFA) in 0.1 M phosphate buffered saline (PBS). The brains were removed and immersion-fixed in the same fixative at 4°C for overnight and 20 μm thick coronal sections were prepared on a cryostat (Leica). After washing in PBS, the sections were blocked in PBS containing 0.3% Triton X-100 and 5% normal goat serum for 1 hr, and then incubated with mouse monoclonal anti-NeuN antibody (Millipore, 1:50) at 4°C overnight. After washing with PBS, the sections were incubated at room temperature for 1 hr with Alexa Fluor 488-conjugated goat anti-mouse IgG (Invitrogen, 1:200) and DAPI (Doujin Kagaku, 1:1000), and the fluorescent signals were detected using a fluorescence microscope (Axiovert 200 M, Carl Zeiss). For quantitative analysis, we examined the thickness of the pyramidal cell layer and the neuron density in five hippocampus sections per mouse. The neuron density was calculated as the number of NeuN immunoreactive cells per 0.01 mm^2^.

### Golgi impregnation

Mice (14 weeks old, male) were perfused through the ascending aorta with 0.15 M NaCl in 0.05 M potassium phosphate buffer (pH 7.4), followed by ice-chilled 1% paraformaldehyde and 1% glutaraldehyde in 0.12 M potassium phosphate buffer (pH 7.4) including 0.75% chloride calcium. Golgi staining was conducted in a manner similar to that previously described [36]. In short, the brains were quickly removed and immersed in 2% Osmium oxide in 0.12 M potassium phosphate buffer (pH 7.4) including 7% glucose in the dark for 24 hr at 4°C. After these fixations, the brains were immersed in 0.2% Osmium oxide, 3.2% potassium dichromate solution for 4 days, and after that, 0.75% silver chloride solution for 4 days. The brains were briefly rinsed with distilled water, dehydrated in a graded ethanol series, and embedded in paraffin wax. Sagittal sections (20 μm) were cut on a sliding microtome, paraffin wax was removed in xylene, and then mounted with Enterannew (Merck Ltd., Japan).

### Spine analysis

Cells with clearly stained basal dendrites and dendritic spines of pyramidal cells were captured under 1000 magnification using photomicroscope DP30 (Olympus, Tokyo, Japan) and digitized using the image analyzing software WINROOF (Mitani Corporation, Fukui, Japan). In the present experiment, at least 15 Golgi stained cells were randomly chosen for quantitative analysis of basal side dendritic spines of hippocampal CA1 pyramidal neurons, from each animal. More than 10 spines per each cell were selected. In total more than 750 spines were measured in each subfield of hippocampus. Measured dendritic spines were located within 50–100 μm distance from the cell body, that correspond to second or third branching of the dendritic projections. The density, length, width, and area of these stained dendritic spines were measured with the image analyzing software WINROOF (Mitani Corporation, Fukui, Japan) combined with photomicroscopy (Additional file 6: Figure S6).

### Conventional thin-section electron microscopy

Mice (10 weeks old, male) were perfused with 2.5% glutaraldehyde in 0.1 M phosphate buffer (pH 7.4). The hippocampi were removed and immersion-fixed in the same fixative at 4°C for 2 hr, and then subjected to post-fixation at 4°C for 1 hr in 1% OsO_4_ in 0.1 M phosphate buffer (ph 7.4). After block staining for 2 hr with 2% uranyl acetate in distilled water, they were dehydrated in graded concentrations of ethanol, incubated with propylene oxide, and embedded in Epok812 epoxy resin. Ultrathin sections were cut with an ultramicrotome (MT-1; Sorval, New Town, Conn. USA) and stained with uranyl acetate and lead citrate. Images were obtained with a transmission electron microscope (JEM-1010; JEOL, Tokyo). For quantitative analysis, we examined the postsynaptic density (PSD) length, thickness and area in the eight synapses that had clearly visible synaptic structures (presynaptic membrane, synaptic cleft, postsynaptic membrane, and PSD) per mouse.

### Electrophysiology

Mice were housed in the institutional standard condition (14:10 light/dark cycle; lights on at 6:00 A.M. through 8:00 P.M.) at 23 ± 1°C with food and water available ad libitum. Mice (8 to 18 weeks old, male) were decapitated under deep halothane anesthesia and both hippocampi were isolated. Transverse hippocampal slices (380 μm) were cut using a tissue slicer in ice-cold standard saline composed of (in mM): NaCl, 125; KCl, 2.5; NaH_2_PO_4_, 1.0; NaHCO_3_, 26.2; glucose, 11; CaCl_2_, 2.5; MgCl_2_, 1.3 (equilibrated with 95% O_2_/5% CO_2_). Slices were then incubated for 30 min at 30°C and maintained in a humidified interface holding chamber at room temperature (24–27°C) before recordings. Electrophysiological recordings were made in a submersion-type chamber maintained at 27.0 - 27.5°C and superfused at 2 ml/min with standard saline. Field excitatory postsynaptic potentials (fEPSPs) were recorded using a glass pipette filled with 2 M NaCl. For recording fEPSPs at the Schaffer collateral/commissural fiber-CA1 synapse, the recording electrode and tungsten bipolar stimulating electrodes were placed in the stratum radiatum in the hippocampal CA1 region. Synaptic potentials were recorded in the saline supplemented with picrotoxin unless otherwise stated. The input–output relationship, paired-pulse facilitation, and PTP were examined in the presence of D-2-Amino-5-phosphonovaleric acid (D-APV), an antagonist of N-methyl-D-aspartate NMDA receptors. LTP and PTP were induced by high-frequency tetanic stimulation (100 Hz, 1 s). In LTP experiments, the tetanic stimulation was delivered after recording stable baseline fEPSPs for at least 20 min. The initial slope of fEPSPs was measured on analysis. Single-pulse stimulation was delivered at a frequency of 0.05 Hz for all baseline recordings. Electrical signals were recorded using a Multiclamp 700B amplifier (Molecular Devices, Sunnyvale, CA, USA), filtered at 2 kHz and stored in a personal computer via an interface (digitized at 5–10 kHz). D-AP5 was purchased from Tocris Bioscience (Bristol, UK). Picrotoxin was from Wako Pure Chemical Industries (Osaka, Japan). All procedures were approved by the Animal Care and Use Committee of Nippon Medical School.

### Analysis of monoamines and their metabolites

Mice (15 weeks old, female) were killed by cervical dislocation. Tissues were homogenized in 10 volumes of buffer comprising 50 mM Tris–HCl (pH 8.0), 100 mM KCl, 0.1 mM EDTA, 1 mM dithiothreitol, 10% glycerol, and protease inhibitors (1 μg/mL pepstatin A, 2 μg/mL leupeptin, and 0.5 μM phenylmethylsulfonyl fluoride). Protein concentration was determined by the method of Bradford [37]. Homogenates were deproteinized with perchloric acid, and contents of monoamines, and their metabolites were analyzed by HPLC-electrochemical detection (ECD-300 system, EICOM, Kyoto).

### Statistical analysis

Behavioral data were analyzed by one-way ANOVA or two-way repeated measures ANOVA. Morphological, biochemical and electrophysiological data were analyzed by two-tailed Student’s t-test. Values in graphs and tables were expressed as mean ± SEM. The number of animals used is indicated by “n” unless otherwise stated. Values of p < 0.05 were considered to indicate statistical significance.

## Abbreviations

APC: Adenomatous polyposis coli; FAP: Familial adenomatous polyposis; PSD: Postsynaptic density; PSD-95: Postsynaptic density-95; LTP: Long-term potentiation; PTP: Post-tetanic potentiation; NMDA: N-methyl-D-aspartate; AMPA: α-Amino-3-hydroxy-5-methyl-4-isoxazolepropionic acid; NeuN: Neuron-specific nuclear protein; PFA: Paraformaldehyde; PBS: Phosphate buffered saline; fEPSP: Field excitatory postsynaptic potential; D-APV: D-2-amino-5-phosphonovaleric acid.

## Competing interests

The authors declare that they have no competing interests.

## Authors’ contributions

TO, KT and TM performed behavioral experiments, KK and NS performed electrophysiological experiments, TO, KS, MK and RN performed morphological experiments, CSI and K Kondo performed analysis of monoamines, RS and RF established *Apc*^*1638T/1638T*^ mice, AS, AIK and TA participated in the improved backcrossing of *Apc*^*1638T/1638T*^ mice, TO, KK, KT and TS drafted the manuscript, KK, TM and TA participated in the design of the study, TS designed and supervised the study. All authors read and approved the final manuscript.

## Supplementary Material

Additional file 1: Figure S1 No significant difference in the hot plate test between *Apc*^*+/+*^ and *Apc*^*1638T/1638T*^ mice. Click here for file

Additional file 2: Figure S2 Distance traveled during footshocks in the training phase of fear conditioning. Click here for file

Additional file 3: Figure S3 Startle response/prepulse inhibition tests. Click here for file

Additional file 4: Figure S4 No significant differences in the thickness of the pyramidal cell layer and the number of NeuN-immunoreactive cells in the hippocampal CA1 region between *Apc*^*+/+*^ and *Apc*^*1638T/1638T*^ mice. Click here for file

Additional file 5: Figure S5 LTP in the hippocampal CA1 region induced in the presence of picrotoxin. Click here for file

Additional file 6: Figure S6 Measuring spine morphology. Click here for file
