# Supplementary material for: Targeted deletion of the C-terminus of the mouse adenomatous polyposis coli tumor suppressor results in neurologic phenotypes related to schizophrenia
Source: Mol Brain. 2014 Mar 29;7:21. doi: 10.1186/1756-6606-7-21 (PMC3986642; doi:10.1186/1756-6606-7-21)
Supplement: Additional file 1: Figure S1 — No significant difference in the hot plate test between Apc+/+ and Apc1638T/1638T mice. [file 1756-6606-7-21-S1.pdf]

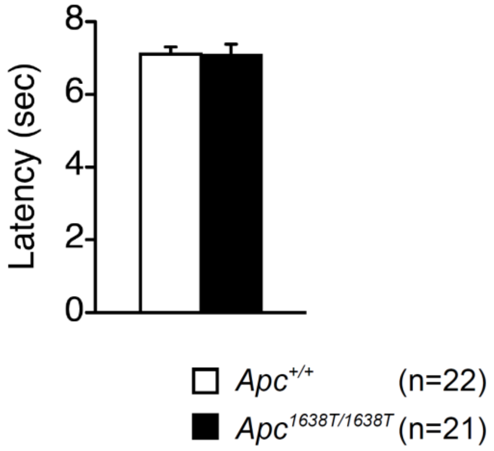

Figure S1 No significant difference in the hot plate test between *Apc*$^{+/+}$ and *Apc*$^{1638T/1638T}$ mice.
