# Supplementary material for: Targeted deletion of the C-terminus of the mouse adenomatous polyposis coli tumor suppressor results in neurologic phenotypes related to schizophrenia
Source: Mol Brain. 2014 Mar 29;7:21. doi: 10.1186/1756-6606-7-21 (PMC3986642; doi:10.1186/1756-6606-7-21)
Supplement: Additional file 2: Figure S2 — Distance traveled during footshocks in the training phase of fear conditioning. [file 1756-6606-7-21-S2.pdf]

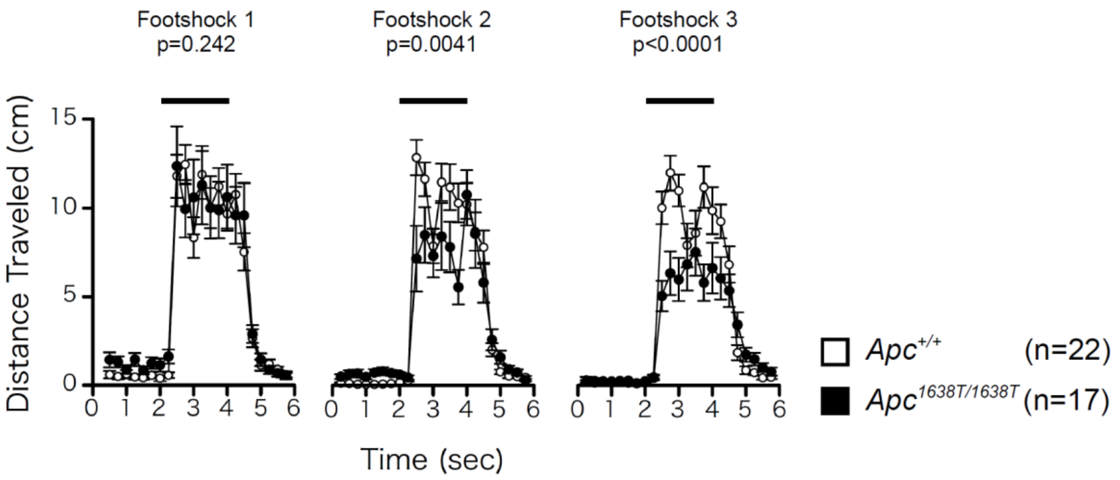

Figure S2 Distance traveled during footshocks in the training phase of fear conditioning. Footshocks were delivered at horizontal bars. The p values indicate genotype effect in two-way repeated measures ANOVA.
