# Supplementary material for: Targeted deletion of the C-terminus of the mouse adenomatous polyposis coli tumor suppressor results in neurologic phenotypes related to schizophrenia
Source: Mol Brain. 2014 Mar 29;7:21. doi: 10.1186/1756-6606-7-21 (PMC3986642; doi:10.1186/1756-6606-7-21)
Supplement: Additional file 3: Figure S3 — Startle response/prepulse inhibition tests. [file 1756-6606-7-21-S3.pdf]

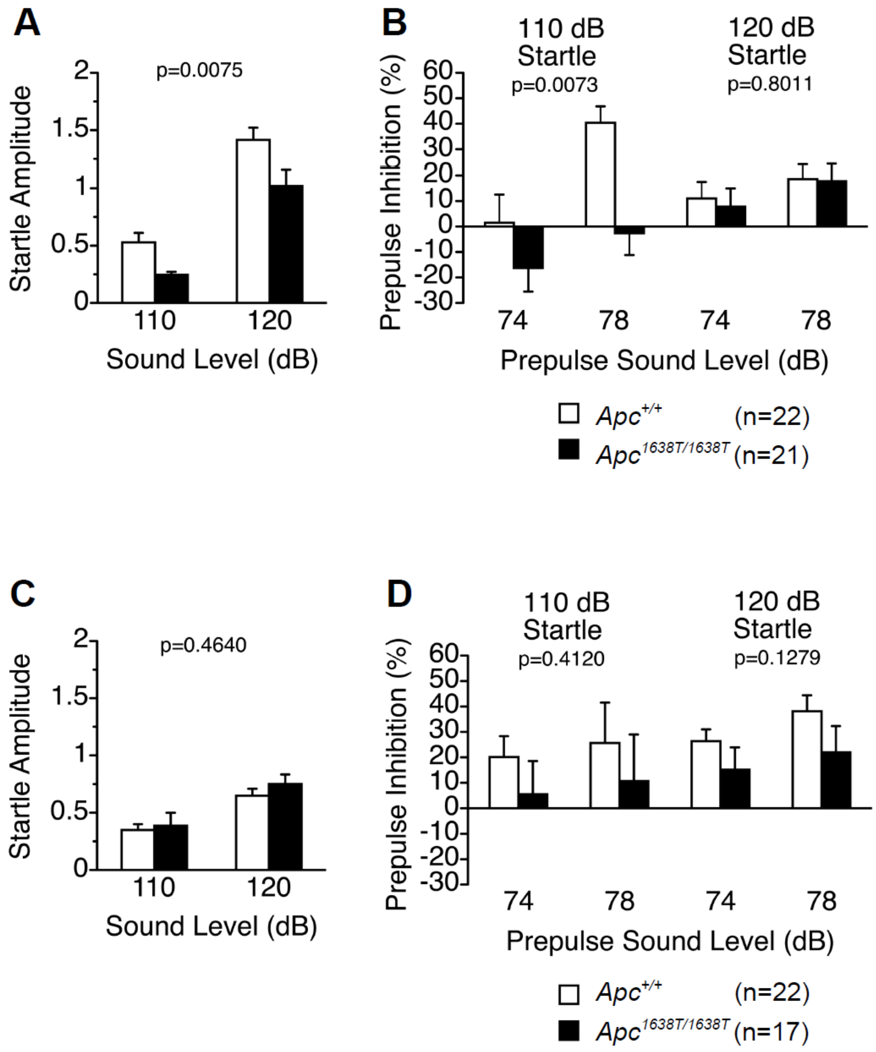

Figure S3 Startle response/prepulse inhibition tests. (A) Startle response and **(B)** prepulse inhibition examined in 13 to 16 weeks old mice. Since prepulse inhibition was not reliably seen in the wild-type mice, the same tests were repeated at 28 to 31 weeks **(C and D)**. The p values in the figure indicate genotype effect in two-way repeated measures ANOVA.
