# Supplementary material for: Targeted deletion of the C-terminus of the mouse adenomatous polyposis coli tumor suppressor results in neurologic phenotypes related to schizophrenia
Source: Mol Brain. 2014 Mar 29;7:21. doi: 10.1186/1756-6606-7-21 (PMC3986642; doi:10.1186/1756-6606-7-21)
Supplement: Additional file 4: Figure S4 — No significant differences in the thickness of the pyramidal cell layer and the number of NeuN-immunoreactive cells in the hippocampal CA1 region between Apc+/+ and Apc1638T/1638T mice. [file 1756-6606-7-21-S4.pdf]

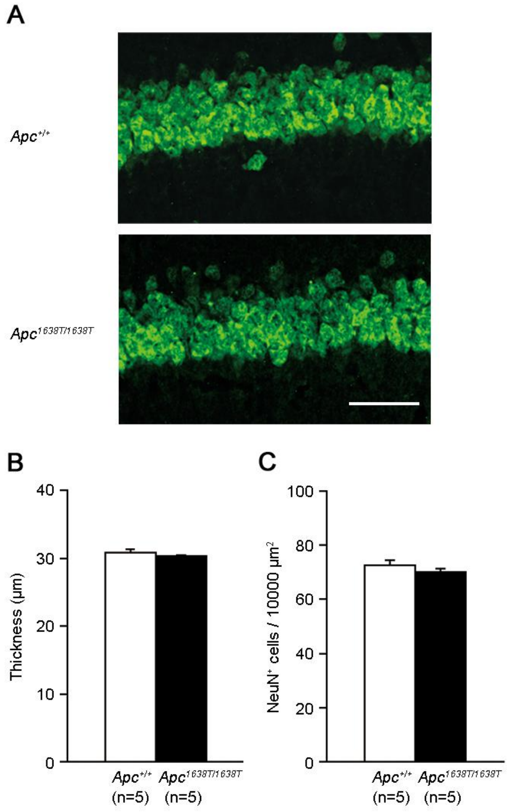

Figure S4 No significant differences in the thickness of the pyramidal cell layer and the number of NeuN-immunoreactive cells in the hippocampal CA1 region between *Apc*$^{+/+}$ and *Apc*$^{1638T/1638T}$ mice. **(A)** Photomicrographs of NeuN-immunoreactive cells in the hippocampal CA1 region. **(B)** Thickness of the pyramidal cell layer in the hippocampal CA1 region. **(C)** Densities of NeuN-immunoreactive cells in the hippocampal CA1 region. Error bars indicate SEM. The scale bar represents 50 μm.
