# Supplementary material for: Targeted deletion of the C-terminus of the mouse adenomatous polyposis coli tumor suppressor results in neurologic phenotypes related to schizophrenia
Source: Mol Brain. 2014 Mar 29;7:21. doi: 10.1186/1756-6606-7-21 (PMC3986642; doi:10.1186/1756-6606-7-21)
Supplement: Additional file 5: Figure S5 — LTP in the hippocampal CA1 region induced in the presence of picrotoxin. [file 1756-6606-7-21-S5.pdf]

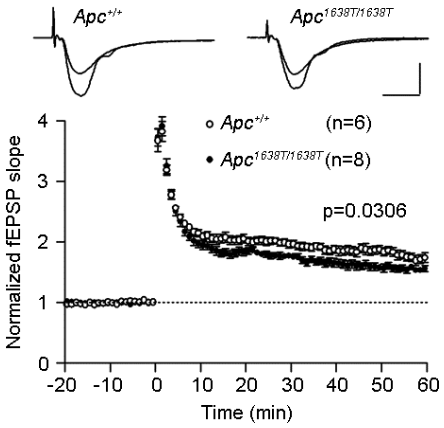

Figure S5 LTP in the hippocampal CA1 region induced in the presence of picrotoxin. As in standard saline, LTP was reduced in the presence of picrotoxin in mutant mice. Scale bars, 10 ms, 0.5 mV. Error bars indicate SEM. The number of slices used is indicated by “n”.
