# Supplementary material for: Targeted deletion of the C-terminus of the mouse adenomatous polyposis coli tumor suppressor results in neurologic phenotypes related to schizophrenia
Source: Mol Brain. 2014 Mar 29;7:21. doi: 10.1186/1756-6606-7-21 (PMC3986642; doi:10.1186/1756-6606-7-21)
Supplement: Additional file 6: Figure S6 — Measuring spine morphology. [file 1756-6606-7-21-S6.pdf]

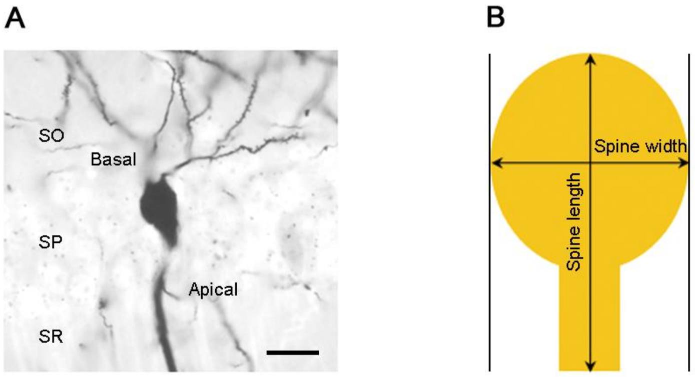

Figure S6 Measuring spine morphology. (A) Golgi-impregnated pyramidal neuron in the hippocampal CA1 region. SO: stratum oriens, SP: stratum pyramidale, SR: stratum radiatum, Basal: Basal side of the pyramidal neuron, Apical: Apical side of the pyramidal neuron. The scale bar represents 20 μm. **(B)** Schematic drawing of a dendritic spine. Length indicates the longest length of the spine. The width is the shortest length between two lines parallel to the longest length of the spine.
